# A Simple and Atraumatic Loop‐Passing Technique for Tibial Anterolateral Ligament Graft Passage in Sonnery‐Cottet Anterior Cruciate Ligament + Anterolateral Ligament Reconstruction

**DOI:** 10.1002/atn2.70305

**Published:** 2026-10-08

**Authors:** Yukio Nakajima, Koichi Muramatsu, Kazue Hayakawa, Yosuke Kaneko, Sho Nojiri, Yusuke Kawano, Nobuyuki Fujita

**Affiliations:** ^1^ Department of Orthopaedic Surgery School of Medicine Fujita Health University Toyoake Japan; ^2^ Department of Orthopaedic Surgery Hachiya Orthopaedic Hospital Nagoya Japan

## Abstract

The purpose of this technical note is to describe a simple and atraumatic loop‐passing technique that facilitates tibial anterolateral ligament (ALL) graft passage in Sonnery‐Cottet combined anterior cruciate ligament and ALL reconstruction. In the Sonnery‐Cottet technique, 2 converging tibial tunnels are created for the ALL, requiring a passing loop to shuttle the graft between tunnels. Conventionally, a needle‐mounted braided suture is advanced blindly through subcutaneous tissue, which can be unreliable in patients with thick subcutaneous tissue and may increase the risk of cortical breach or extraosseous passage. We describe a knot‐pusher‐assisted loop‐passing technique using a 0 silk suture. A knot pusher loaded with the silk suture is introduced through 1 tibial tunnel and positioned at the tunnel convergence zone. The silk loop is retrieved from the second tunnel using a probe, creating a continuous loop for graft passage. This technique provides a simple, reliable, and atraumatic method for tibial ALL graft passage in Sonnery‐Cottet combined anterior cruciate ligament and ALL reconstruction.

## Graphical abstract

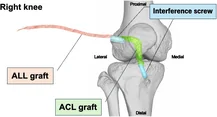

**VIDEO 1:** Knot‐pusher‐assisted loop‐passing technique for tibial anterolateral ligament graft passage in Sonnery‐Cottet combined anterior cruciate ligament and anterolateral ligament reconstruction. The video shows controlled blind loop retrieval within converging tibial tunnels using a probe, creation of a continuous passing loop, and reliable atraumatic graft shuttling without blind needle advancement. Video content can be viewed at https://doi.org/10.1002/atn2.70305.

Lateral extra‐articular procedures have gained increasing attention as an adjunct to anterior cruciate ligament (ACL) reconstruction to improve rotational stability and reduce the risk of graft failure and secondary meniscal procedures.^1^, ^2^ Several recent studies have showed that residual anterolateral rotatory instability after isolated ACL reconstruction may contribute to persistent pivot‐shift phenomenon and inferior clinical outcomes. Consequently, the addition of an extra‐articular reinforcement has been proposed, particularly in young, active patients, revision cases, and individuals with high‐grade pivot shift.^3^

Among various lateral extra‐articular procedure techniques, anterolateral ligament (ALL) reconstruction has been widely adopted. Sonnery‐Cottet et al. described a combined ACL and ALL reconstruction technique (S‐C technique), which has showed favorable mid‐ to long‐term clinical outcomes.^4^ This technique reproduces the native differential length‐change pattern of the ALL,^5^ shares a common femoral tunnel with the ACL graft to avoid tunnel convergence, and does not require additional implants for ALL fixation, thereby maintaining a low‐cost profile.

Despite these advantages, the S‐C technique has a technical limitation related to graft passage on the tibial side of the ALL. Two tibial tunnels must be connected internally, and a passing loop is required to shuttle the graft between them.^6^ Conventionally, a needle‐mounted braided suture is used; however, this step may be technically demanding in patients with thick subcutaneous tissue and may increase the risk of extraosseous passage or cortical breach.

The purpose of this technical note is to describe a simple, reliable, and atraumatic loop‐passing technique using a knot pusher that facilitates tibial ALL graft passage during the S‐C combined ACL and ALL reconstruction.

## SURGICAL TECHNIQUE

### Indications and Patient Positioning

This technique is indicated for patients undergoing combined ACL and ALL reconstruction using the S‐C method. Indications for adding ALL reconstruction include high‐grade pivot‐shift phenomenon, participation in pivoting sports, revision ACL surgery, generalized ligamentous laxity, and young age. The patient is placed in the supine position with a lateral post to allow valgus stress during arthroscopy. Standard anterolateral and anteromedial portals are established for ACL reconstruction. All steps of intra‐articular ACL reconstruction are performed according to the surgeon's routine protocol before proceeding to the extra‐articular ALL reconstruction (Figure 1).

**FIGURE 1:**
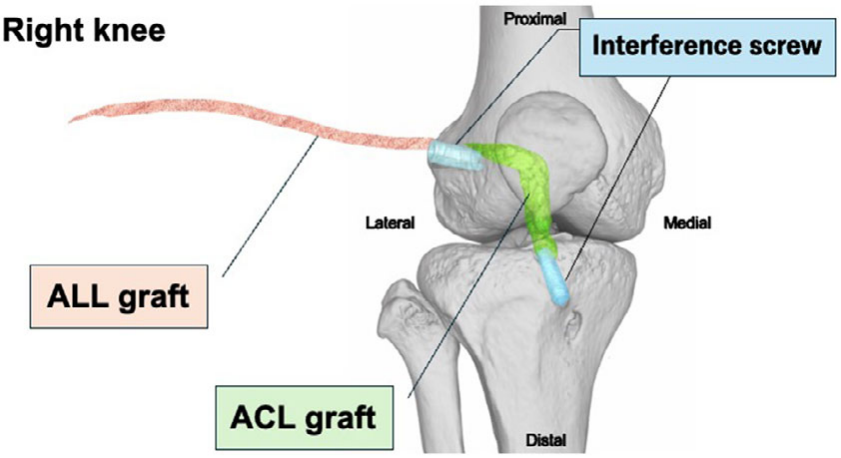
Final configuration after ACL reconstruction and preparation for ALL graft passage in a right knee. Complete ACL reconstruction fixed with an interference screw. The gracilis tendon, serving as the ALL graft, is seen exiting the femoral tunnel. (ACL, anterior cruciate ligament; ALL, anterolateral ligament.)

### Creation of Tibial ALL Tunnels^6^

Two tibial tunnels for the ALL are created according to the standard S‐C technique. The proximal tunnel is drilled at the anatomic tibial insertion of the ALL, located between Gerdy's tubercle and the fibular head. The distal tunnel is drilled more posteriorly, and both tunnels are oriented to converge internally within the tibia (Figures 2 and 3). This configuration allows the ALL graft to be passed from 1 tunnel to the other, reproducing the physiologic course of the ligament. Care is taken to maintain adequate bone stock and to avoid tunnel convergence with the ACL tibial tunnel. After tunnel creation, attention is directed to the preparation of a passing loop for graft shuttling.

**FIGURE 2:**
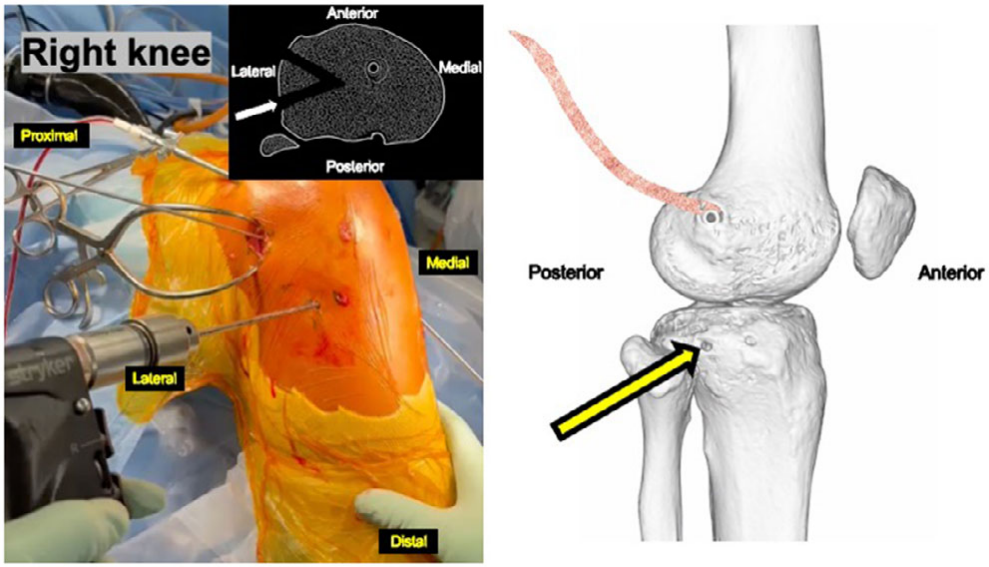
Creation of the first tibial tunnel for ALL reconstruction in a right knee. Two tibial tunnels for the ALL are created using a 4‐mm drill. The first tunnel is positioned at the level of the anterior border of the fibular head, approximately 1.5 cm distal to the joint line. (ALL, anterolateral ligament.)

**FIGURE 3:**
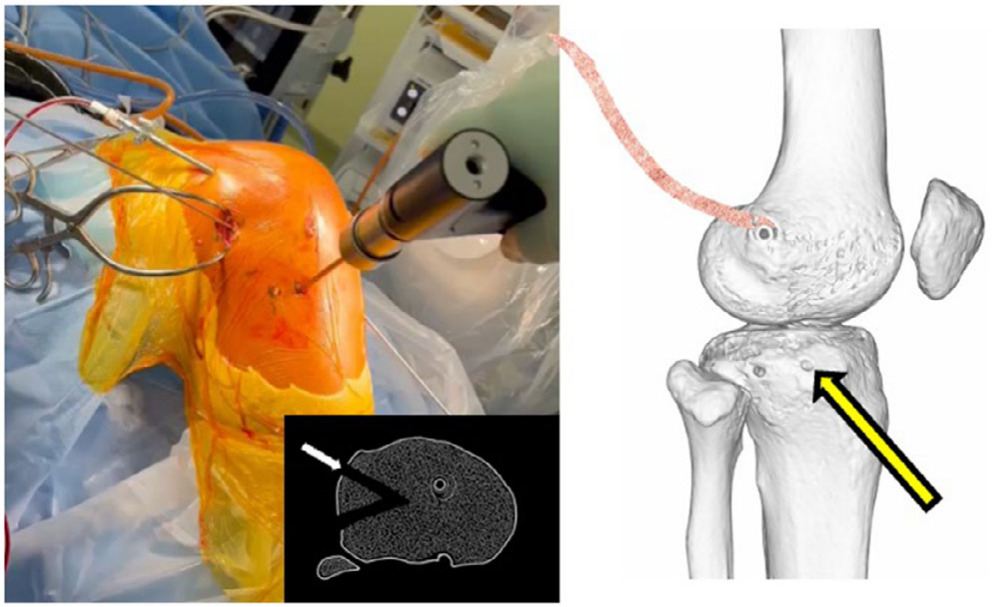
Creation of anterior tibial tunnels for anterolateral ligament reconstruction of a right knee. The second tunnel is similarly created at the level of the posterior border of Gerdy's tubercle. Both tunnels are drilled to converge and communicate within the tibia.

### Conventional Method and Its Limitations

Traditionally, a looped suture is passed through the tibial ALL tunnels using a needle‐mounted braided suture, such as No. 5 Ethibond (Ethicon, Somerville, NJ). The needle is reversed and introduced through 1 tunnel, advanced blindly through subcutaneous tissue, and retrieved from the second tunnel using a probe or mosquito clamp. This maneuver can be technically demanding, particularly in patients with thick subcutaneous fat layers. In such cases, the needle may fail to reach the tunnel, resulting in inadvertent extraosseous passage. Additionally, forceful manipulation may cause cortical breach or enlargement of the tunnel entrance. These limitations may prolong operative time and compromise the reproducibility of the S‐C technique.

### Knot‐Pusher‐Assisted Loop‐Passing Technique

#### Preparation

A 0 silk suture (Alfresa Pharma, Osaka, Japan) is threaded through the lumen of a standard arthroscopic knot pusher, creating a free loop at the distal end. Both free ends of the silk suture remain outside the handle of the knot pusher (Video 1).

#### Insertion

The knot pusher is introduced through 1 of the tibial ALL tunnels and advanced until its distal tip reaches the convergence zone of the 2 tunnels (Figure 4). The rigid shaft of the knot pusher allows precise control of the distal tip without blind advancement through subcutaneous tissue.

**FIGURE 4:**
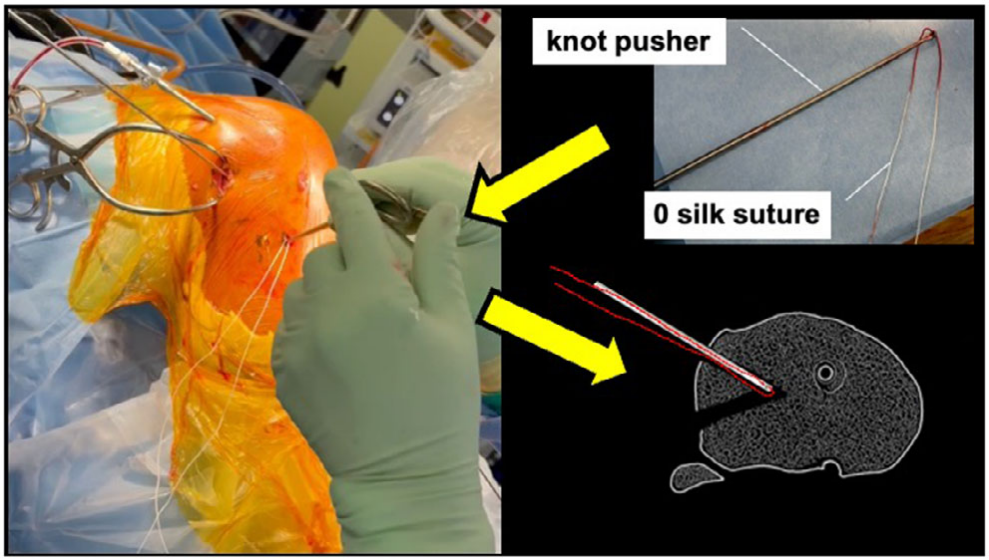
Insertion of the knot pusher loaded with a silk suture into the anterior tibial tunnel in a right knee. A 0 silk suture is threaded through the distal eyelet of a knot pusher, which is then inserted into the anterior tibial tunnel.

#### Retrieval

Through the second tibial tunnel, a probe is inserted and used to hook the silk loop protruding from the knot pusher tip (Figure 5). The silk suture is gently pulled out through the second tunnel while maintaining the position of the knot pusher (Figure 6).

**FIGURE 5:**
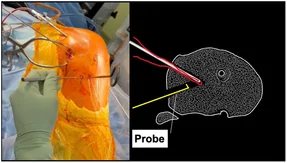
Advancement of the knot pusher to the tunnel convergence zone and introduction of the probe in a right knee. The knot pusher is advanced to the tunnel convergence zone within the tibia, after which a probe is introduced through the posterior tibial tunnel.

**FIGURE 6:**
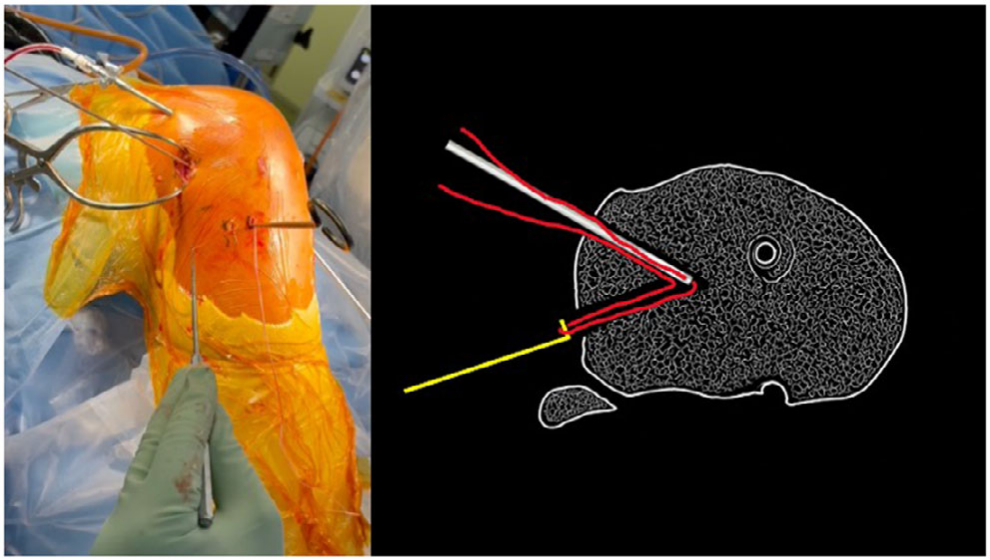
Probe‐assisted retrieval of the silk suture to establish a continuous loop in a right knee. The probe is advanced through the posterior tibial tunnel to the convergence zone within the tibia. While carefully maintaining awareness of the probe tip orientation, the silk suture attached to the knot pusher is engaged with the probe and gently retrieved through the posterior tunnel.

#### Loop Creation

Once the silk suture is retrieved, a continuous loop is established between the 2 tibial tunnels. The knot pusher is then removed, leaving the silk loop securely in place.

#### Graft Passage

The prepared ALL graft is attached to the silk loop and shuttled through the tibial tunnels in a controlled manner. After successful passage, the graft is fixed on the femoral side and tied according to the standard S‐C technique (Figure 7). This method avoids blind needle advancement, minimizes the risk of tunnel damage, and allows consistent loop creation regardless of patient body habitus.

**FIGURE 7:**
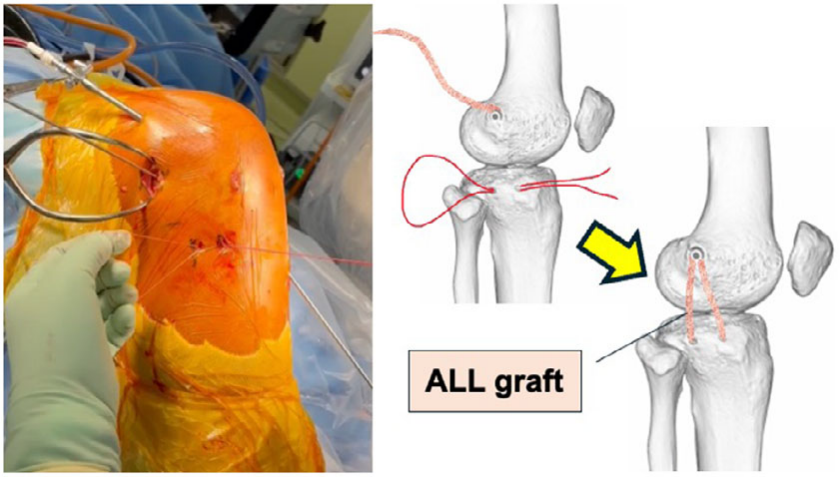
Graft passage and completion of ALL reconstruction in a right knee. A continuous loop for graft passage is established between the tibial tunnels. The ALL graft is then passed beneath the ITB and shuttled through the tibial tunnels using the prepared loop. The graft is subsequently routed back to the femoral side along the same layer and secured with knot tying, completing the ALL reconstruction. (ALL, anterolateral ligament; ITB, iliotibial band.)

### Ethical Approval

This technical note was approved by the institutional review board of our institution (Approval No. HM23‐357Hhm). Written informed consent was obtained from all patients.

## DISCUSSION

The S‐C combined ACL and ALL reconstruction technique offers several advantages, including restoration of the anterolateral stabilizing structures, avoidance of femoral tunnel convergence, and elimination of additional implants for ALL fixation. These characteristics make it an attractive and cost‐effective procedure. However, its reproducibility may be compromised by technical difficulties associated with tibial graft passage.

The conventional needle‐based loop‐passing technique relies on blind advancement of a needle‐mounted suture through subcutaneous tissue. This maneuver may be unreliable in patients with increased subcutaneous fat thickness and may lead to extraosseous suture passage, cortical breach, or tunnel enlargement. Such complications can prolong operative time and increase the technical complexity of the procedure.

The knot‐pusher‐assisted technique described in this study provides several advantages. First, the rigid shaft of the knot pusher allows precise control of the instrument tip, enabling accurate placement within the tunnel convergence zone without blind advancement. Second, the use of a silk suture loop facilitates atraumatic retrieval with a probe, minimizing stress on the cortical bone. Third, this method requires only commonly available instruments and maintains the low‐cost advantage of the S‐C technique.

Another important advantage is its applicability in obese patients. In such individuals, conventional needle‐based techniques may fail because of insufficient needle length. The knot‐pusher‐assisted method bypasses this limitation and provides a consistent and reproducible means of loop creation regardless of patient body habitus (Table 1).

**TABLE 1:** Advantages and Disadvantages of the Knot‐Pusher‐Assisted Loop‐Passing Technique

**Advantages**	**Disadvantages**
Avoids blind needle advancement through subcutaneous tissue	Requires familiarity with arthroscopic instruments (knot pusher and probe)
Enables controlled and precise positioning within the tunnel convergence zone	Blind retrieval within the tunnel may require practice
Reduces risk of extraosseous suture passage	Dependent on adequate tunnel convergence
Minimizes risk of cortical breach and tunnel enlargement	Limited evidence because of the technical note nature
Provides consistent loop creation regardless of subcutaneous thickness	No comparative clinical or biomechanical validation
Uses commonly available instruments without additional implants	
Maintains the low‐cost nature of the S‐C technique	

S‐C, Sonnery‐Cottet.

This technique has several limitations, including its descriptive nature and lack of a formal comparative analysis. No biomechanical testing or clinical outcome comparisons were performed between the knot‐pusher‐assisted technique and conventional needle‐based methods. Additionally, the results reflect the experience of a single institution. Multicenter studies and randomized comparisons are necessary to further validate the generalizability and potential benefits of this technique (Table 2).

**TABLE 2:** Pearls and Pitfalls of the Knot‐Pusher‐Assisted Loop‐Passing Technique

**Pearls**	**Pitfalls**
Mark the handle of the probe to maintain constant awareness of the tip orientation during loop retrieval within the tunnel	Loss of awareness of the probe tip orientation may hinder accurate loop engagement
Use a probe to gently hook the silk loop under controlled conditions	Forceful probing may damage the tunnel or soft tissue
Ensure the silk loop is clearly visualized before retrieval	Failure to identify the loop may prolong operative time
Use smooth traction when retrieving the silk suture	Excessive traction may disrupt loop formation
Confirm a continuous loop before graft passage	Incomplete loop may lead to graft passage failure
Select appropriate tunnel orientation to ensure communication	Poor tunnel alignment may prevent loop creation

The knot‐pusher‐assisted loop‐passing technique provides a simple, reliable, and atraumatic method for tibial ALL graft passage in the S‐C combined ACL and ALL reconstruction. It improves procedural reproducibility, minimizes the risk of tunnel damage, and can be applied regardless of patient body habitus, thereby enhancing the safety and efficiency of this procedure.

## DISCLOSURES

The authors (Y.N., K.M., K.H., Y.K., S.N., Y.K., N.F.) declare that they have no known competing financial interests or personal relationships that could have appeared to influence the work reported in this paper.
